# Impacts of Aggregate Gradation on the Volumetric Parameters and Rutting Performance of Asphalt Concrete Mixtures

**DOI:** 10.3390/ma15144866

**Published:** 2022-07-13

**Authors:** Weihua Li, Weidong Cao, Xianfu Ren, Shurong Lou, Shutang Liu, Jizhe Zhang

**Affiliations:** 1 Shandong High Speed Group Weifang Development Co., Ltd., Weifang 262500, China; wfglsj@163.com (W.L.); renxianfu-001@126.com (X.R.); sdgs144@163.com (S.L.); 2 School of Qilu Transportation, Shandong University, Jinan 250002, China; jizhe.zhang@sdu.edu.cn

**Keywords:** aggregate gradation, asphalt concrete mixture, volumetric parameters, rutting performance, skeletal dense structure

## Abstract

The main objective of this study was to determine the effect of aggregate gradation (AG) on the volumetric parameters (VPs) and rutting performance (RP) of asphalt concrete (AC) mixtures. The boundary sieve (BS) between fine and coarse aggregates was 2.36 mm size, and 15 gradation curves of three nominal maximum aggregate sizes (13.2, 19.0, and 26.5 mm) were designed based on the percentage passing of the BS. A vibrating compaction test of coarse aggregates, Marshall compaction and wheel-tracking tests of AC mixtures with various gradations were conducted. It was found that AG had crucial effects on the VPs and RP of AC mixtures. The AC mixture can be designed as a skeletal dense structure provided that the percentage passing of the BS is appropriate. More notably, AC mixtures with a skeletal dense structure showed the best rutting resistance performance. Therefore, it is important to optimize AG for enhancing the high-temperature RP of AC mixtures.

## 1. Introduction

It is well accepted that the performance of asphalt concrete (AC) pavement is greatly influenced by its aggregate characteristics, since AC mixtures contain approximately 90–95% of mineral aggregate by weight. In particular, aggregate gradation (AG) is a significant factor to be considered in the design of AC mixture. Hence, different methods have been developed and applied on gradation selection [1,2], including Superpave and Marshall mix designs, which meet the requirements for the volumetric properties of AC mixture. The paramount volumetric properties of a mix design include voids in the mineral aggregate (VMA), voids filled with asphalt (VFA), and air voids (AV). Current VMA requirements are built upon the premise that pavement performance and durability reduce when the VMA drops below the minimum value. However, an increase in minimum VMA can attenuate the performance of some mixtures. AG plays an important role in ensuring that an adequate amount of VMA is obtained to achieve the desired performance levels [3]. Therefore, VMA has a crucial effect on the designs of aggregate gradation [4]. According to the Asphalt Institute, the recommendations for optimizing the gradation curves to achieve the desired effects on AV and VMA are largely dependent on the experience of designers in handling different materials [5]. A growing body of research has focused on the association between AG and Maximum Density Line (MDL) and how this connection can affect the VMA of AC mixtures [6].

With regard to volume fraction, the fine and coarse aggregates account for 85–90% of the volume of hot mix asphalt (HMA) [7,8], in which the proportion of coarse aggregates (particle size > 2.36 mm) is also prominent. From a different perspective, coarse aggregates in HMA play dual roles: (i) the internal resistance of HMA is characterized as stone-on-stone skeleton established in the mix design (also for open-graded friction or stone mastic asphalt course), and the coarse aggregate contact of HMA gradation is regarded as the primary source of internal resistance [8,9]; (ii) the percentage voids in the coarse aggregate of asphalt mixture (VCA_mix_) structured by coarse aggregates can provide a maternal space for VMA and partially restrict the VMA values [4]. Hence, VCA_mix_ is also a critical volume indicator that has no direct relationship with the gradation design.

AG is one of the important properties of HMA, as it has various aspects of mixture performance, including VMA, resistance to permanent deformation, durability and compatibility [10,11,12,13,14]. It has been reported that AG is closely related to the rutting and indirect tensile (IDT) performance. The gradation effect is aggregate specific for rutting. The design binder content can be used to measure the effect of gradation on indirect tensile strength (ITS), which is associated with the VMA values of AG [15]. Sangsefidi and colleagues [16] assessed the moisture susceptibility of warm mix asphalt and the effect of AG on creep. Their findings demonstrated that AG could differently affect the moisture vulnerability and rutting resistance of the studied mixtures. Hafeez and co-workers [17] evaluated the effects of AG with various nominal maximum aggregate sizes on the fatigue, rutting and stiffness performance of stone mastic asphalt (SMA). Their findings showed that stiffer SMA had lower rut values and fatigue life. A mixture of stiffness and rut resistance may enhance with increasing aggregate sizes during AG. Xiao and colleagues [18] found that AG could affect the rutting resistance and moisture susceptibility of open graded friction course (OGFC) mixtures. Kim and co-workers [19] conducted a study to examine the effects of mix gradations related to the Superpave restricted zone on rutting potential, particularly for low traffic volume roadways. They concluded that similar to that for medium to high traffic volume pavements, the restricted zone is not a significant factor influencing the RP of HMA for low traffic volume local pavements. The fineness of AG, rather than the restricted zone, may serve as a promising factor that influences rutting performance. Sun et al. [20] evaluated the skeleton contact stability of a graded aggregate system and analyzed the slip creep properties of asphalt mixture from the geometric characteristics of aggregates. Devulapalli et al. [21] summarized the concerns in the SMA mixtures and gave knowledge about the gradation, stone-on-stone contact, drain down, and stabilizing agents based on a detailed literature review.

According to the relevant literature [7] and our research [22,23], a 2.36 mm size can be employed as the boundary sieve (BS) between fine and coarse aggregates. Hence, the coarse aggregates > 2.36 mm constitute the skeleton structure of asphalt mixtures. The AC mixture has been widely used in the asphalt pavement structure in China, which is often regarded as a dense-suspended gradation structure. However, the dense skeleton structure in AC mixture can also be formed when the suitable AG is designed. The main objective of this study was to assess the quantitative impacts of AG variations (i.e., different percentage passing of 2.36 mm sieve) on the volumetric parameters (VPs) and high-temperature RP of AC mixtures. In this paper, the relations between key volumetric parameters, rutting resistance performance and percent passing of BS for AC-13, AC-20 and AC-25 were established, respectively. In addition, the reference values of the percentage passing of BS were presented for guiding the gradation design of AC mixture with dense skeleton structure.

## 2. Materials and Experiments

### 2.1. Materials

#### 2.1.1. Aggregates and Filler

AC mixtures with 3 nominal maximum sizes of 26.5, 19 and 13.2 mm, namely, AC-25, AC-20 and AC-13, were employed in the present study. AC-25, AC-20 and AC-13 were applied in the bottom, middle and upper surface layers of the asphalt pavement, respectively. Considering the mechanical properties and abrasion resistance of crushed basalt stones, they were employed for fine and coarse aggregates in AC-13. Meanwhile, the crushed lime stones were applied for fine and coarse aggregates in AC-25 and AC-20. One basalt fine aggregate (0–3 mm) and three basalt coarse aggregates with different particle sizes (3–5, 5–10 and 10–15 mm) were selected. Table 1 shows the properties of basalt coarse aggregates according to the Chinese specifications [24], while Table 2 shows the properties of lime coarse aggregates with different particle sizes (3–5, 5–10, 10–20 and 20–30 mm). The basic properties of the two kinds of fine aggregates are presented in Table 3. The limestone powder was used as a mineral filler after passing through the #200 sieve. All the materials have met the technical requirements specifications [24].

#### 2.1.2. Asphalt Binder

A 70 penetration grade (Pen 70) asphalt binder was supplied by a commercial petroleum company and was employed in all mixture designs. Table 4 shows the properties of the Pen 70 asphalt binder. The specifications for pavement asphalt binders are met for all data [24].

### 2.2. Mix Design

**Figure 1:**
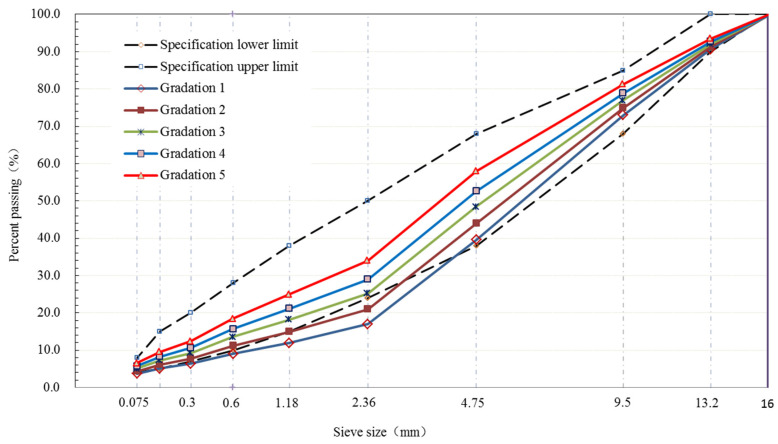
Gradation curves of AC-13.

**Figure 2:**
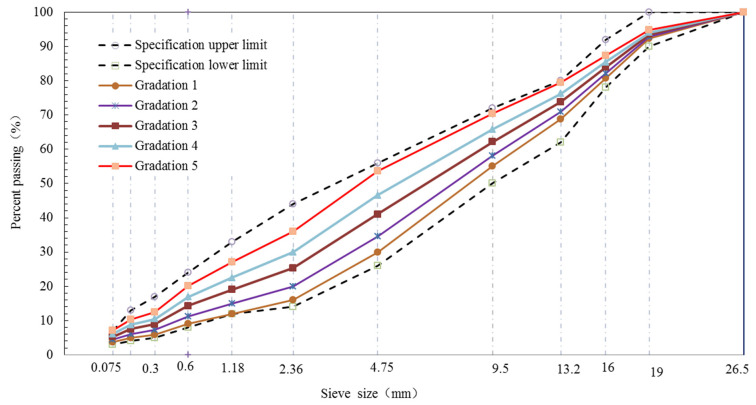
Gradation curves of AC-20.

**Figure 3:**
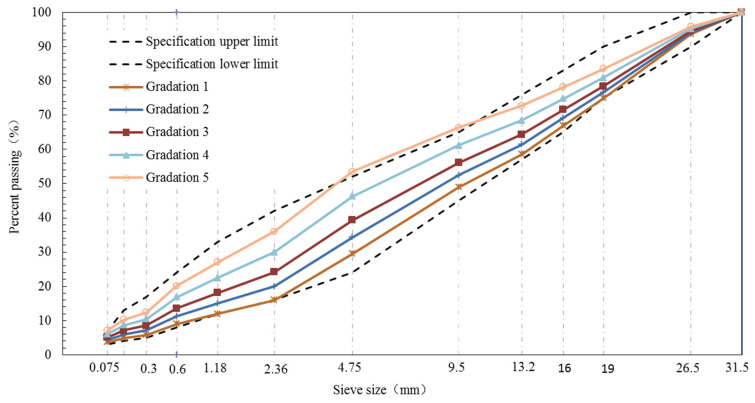
Gradation curves of AC-25.

The size of BS between fine and coarse aggregates was 2.36 mm. Five grading curves of AC-25, AC-20 and AC-13 were designed based on the percent passing (PP) of the BS. The structure and type of the aggregate can be reflected by the PP of the BS. The gradation curves of all aggregates demonstrated S type. Figure 1, Figure 2 and Figure 3 show the design gradation curves of aggregates with 3 nominal maximum sizes. The gradation curves can be seen in two parts consisting of a coarse section (sieve size is equal or greater than 2.36 mm) and fine section (sieve size is less than or equal to 2.36 mm). Additionally, the coarse sections of five gradation curves for each nominal maximum aggregate size were designed as *S* type curves, and the proportion of the coarse aggregates was constant.

According to the China specifications [24], our previous research and field experience of AC mixture [25], the asphalt contents of the three AC mixtures were evaluated, which were 3.9%, 4.4% and 5.3% for AC-25, AC-20 and AC-13, respectively. To further assess the individual effect of AG on the VPs and high-temperature RP of AC mixtures, 5 AGs of each AC mixture used the similar asphalt contents.

### 2.3. Experimental Program and Testing Methods

First of all, VCA formed from the packing of coarse aggregates for three kinds of AC mixtures at the state without any asphalt binders were conducted using vibrating compaction. Then, the VPs and RP of AC mixtures were assessed by determining the effects of 5 levels of AG and 3 levels of nominal maximum aggregate size. A total of 15 AC mixtures were designed with the Marshall method and examined using the two-wheel laboratory tracking devices.

#### 2.3.1. Vibrating Compaction Test of Coarse Aggregates

**Figure 4:**
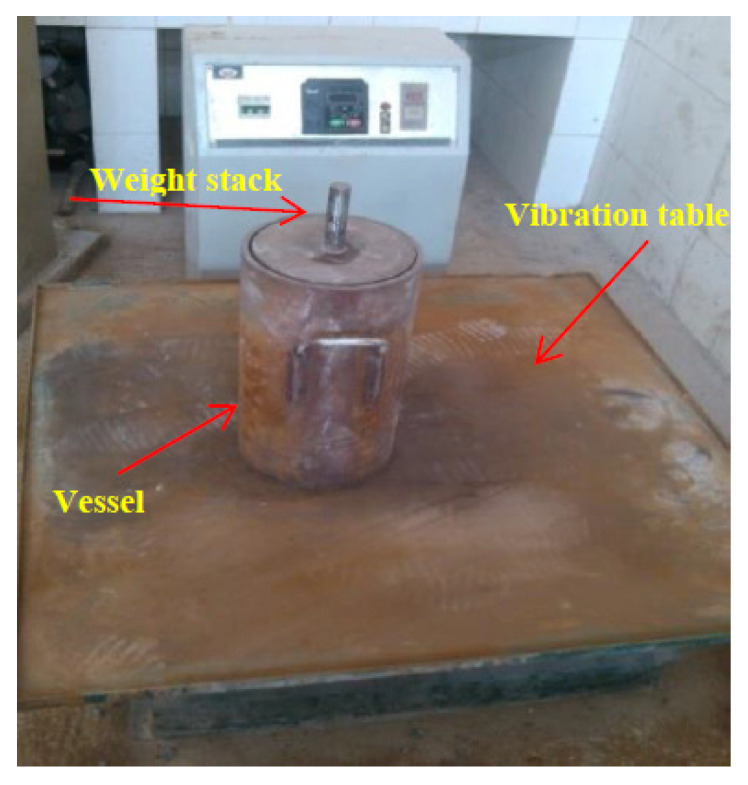
Vibrating compaction test device.

The vibrating compaction test of coarse aggregates was performed by a vibration table, and the vessel with a weight stack is shown in Figure 4. Firstly, different size coarse aggregates were fully mixed by the proportion according to the coarse sections of designed gradation curves, and the mixed specimen was obtained. Next, the mixed specimen was placed in a 10 L capacity vessel 3 times. After loading the sample into a layer, the vessel was placed onto a vibration table, and the compaction experiment was conducted (the vibration frequency and time were 50 Hz and 90 s, respectively). Lastly, when the final layer was vibrated, the surface of the samples was leveled. To maintain data accuracy, the experimental errors were reduced and the number of parallel experiments was set to 3. The values of VCA can be calculated using Equation (1).

(1) $$\mathrm{VCA}=\left(1-\frac{\rho }{\rho_{b}}\right)\times 100$$

where *ρ* is the packing density of the mixed coarse aggregates in the vessel; *ρ_b_* is the synthetic bulk density of coarse aggregates with different sizes.

#### 2.3.2. Marshall Compaction Test

To assess the effects of different AGs on the VPs of AC mixtures, the samples were fabricated with 75 blows of Marshall Compactor. The asphalt mixture samples were prepared according to the T072-2011 [26]. Four samples were fabricated for each AG. The VMA, AV and VCA_mix_ of the compacted specimens of various AGs were tested and calculated in terms of the procedures and formulas described in the China Standard Test Methods [26].

#### 2.3.3. Wheel-Tracking Test

**Figure 5:**
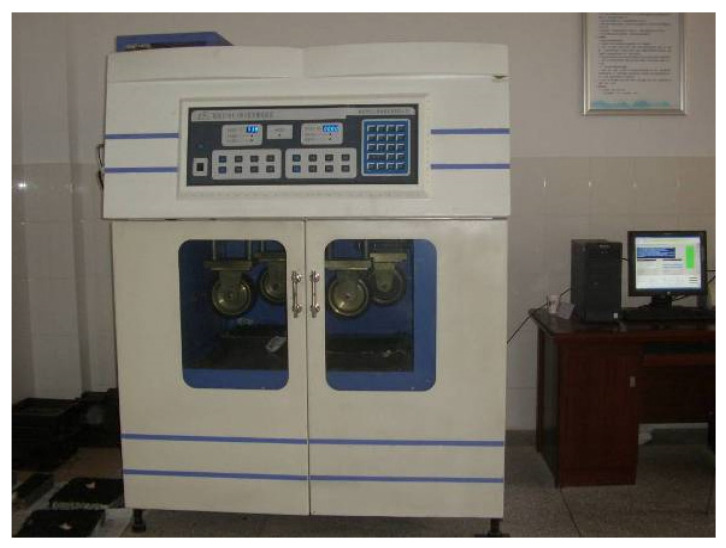
Wheel-tracking test device.

To evaluate the high-temperature RP of the AC mixture, the wheel-tracking test was performed by utilizing the wheel-tracking device (Figure 5). Each sample was 50 mm in height and 300 mm × 300 mm in cross-sectional areas. According to the China Standard T0719-2011 [26], the wheel-tracking test was conducted using the 0.7 MPa wheel load at 60 °C temperature under dry conditions. Dynamic stability (DS) is presented by the number of times the wheel passes the sample per rut depth within an interval of 45–60 min, which can be calculated using Equation (2) [26]:

(2) $$\mathrm{DS}=\frac{\left(t_{2}-t_{1}\right)\times N}{d_{2}-d_{1}}$$

where *d*_1_ and *d*_2_ are rut depth at *t*_1_ (45 min) and *t*_2_ (60 min), respectively; *N* is the speed of wheel passing over the center of the sample, 42 cycles/min.

A high DS of the AC mixture is indicative of an excellent performance of its resistance to permanent deformation at high temperature.

## 3. Results and Discussion

### 3.1. Results of Vibrating and Marshall Compaction Tests

**Figure 6:**
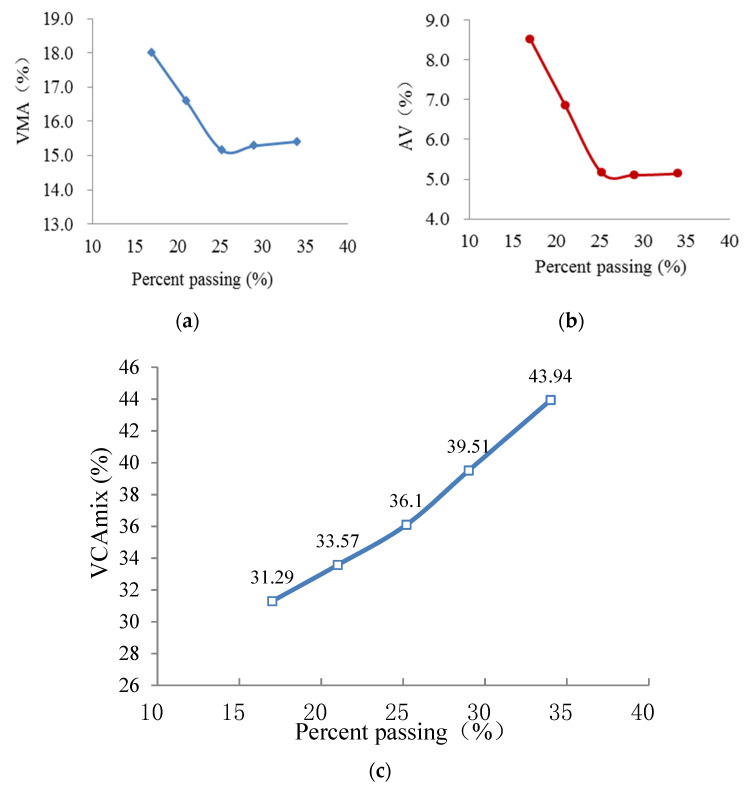
VPs of AC-13 samples versus PP. (**a**) VMA; (**b**) AV; (**c**) VCA_mix_.

**Figure 7:**
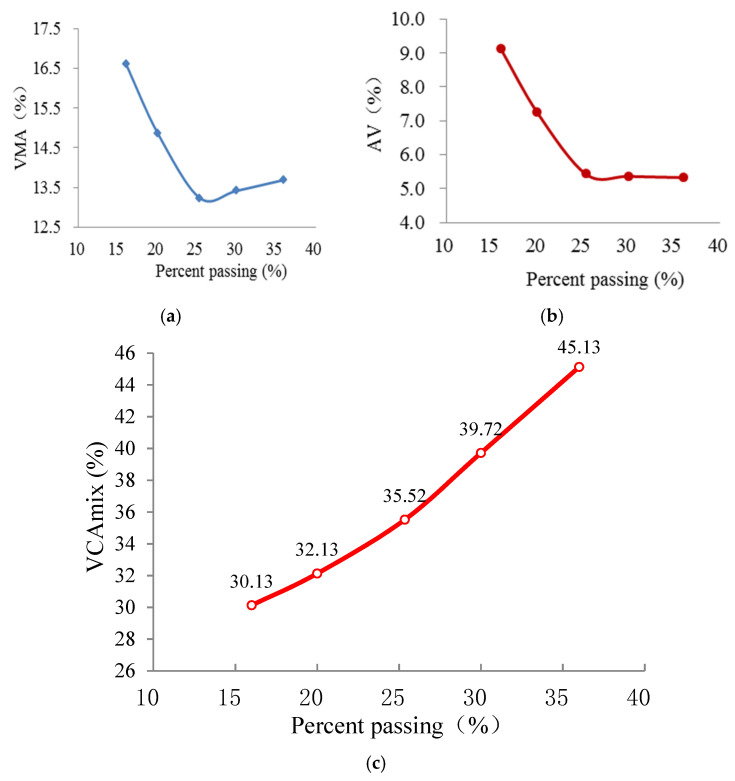
VPs of AC-20 samples versus PP. (**a**) VMA; (**b**) AV; (**c**) VCAmix.

**Figure 8:**
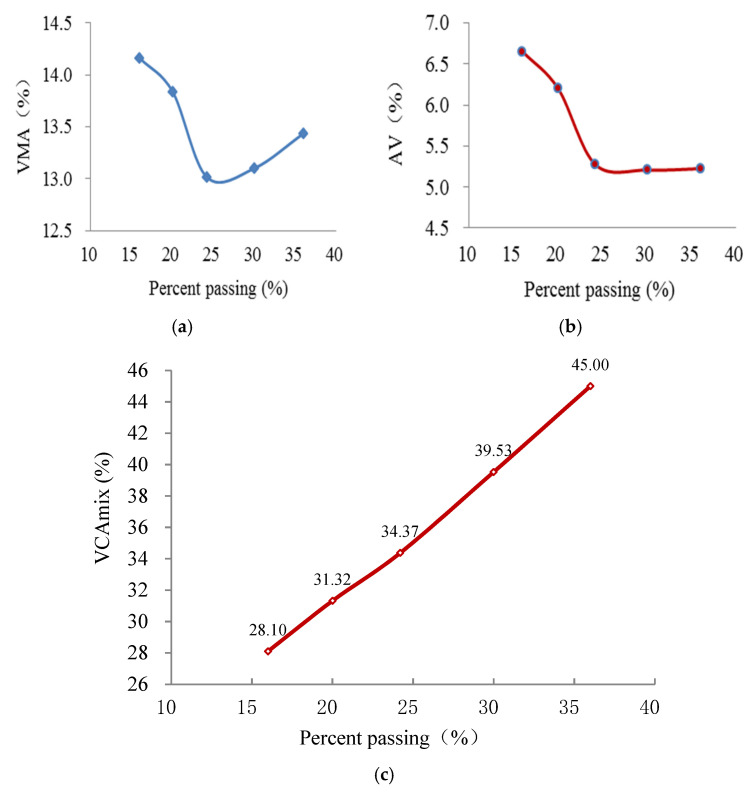
VPs of AC-25 samples versus PP. (**a**) VMA; (**b**) AV; (**c**) VCAmix.

The proportions of various coarse aggregates in the three AC mixes and corresponding VCA values are shown in Table 5. Vibrating compaction tests were repeated three times for each AC mixture, and the values of VCA are represent as means. Based on the testing density parameters of Marshall compacted samples for the three AC mixtures, the calculated results of VMA, AV and VCA_mix_ as a function of the PP of BS are presented in Figure 6, Figure 7 and Figure 8. The average values of the four duplicates are obtained.

As shown in Figure 6, Figure 7 and Figure 8, the AG has crucial effects on the VPs (i.e., VMA, AV and VCA_mix_) of AC mixtures. The values of VMA first decline and then elevate with increasing percentage passing of the BS, and they exhibit an upward-opening parabola, whereas those of AV reduce monotonically and appear to be constant with the increasing percentage passing of the BS. The values of VCA_mix_ show a linear increase with the increasing percentage passing of the BS. In addition, the VPs of different AGs of AC-25, AC-20 and AC-13 have the same rule of change. VMA and AV are close to the smallest value when the PP of BS is around 25. It is speculated that the aggregate structure of the AC mixture changes as a function of the PP of the BS. The inner forms of AC mixtures are skeleton-gap structures when the content of the coarse aggregate is larger (the PP is <25). Meanwhile, the coarse skeleton structures of AC mixtures are disrupted by excessive fine aggregate when the content of fine aggregate is larger (the PP is >25). Hence, the aggregate skeleton structure is in its densest state when the proportion of fine and coarse aggregates is appropriate.

### 3.2. Results of Wheel-Tracking Tests

**Figure 9:**
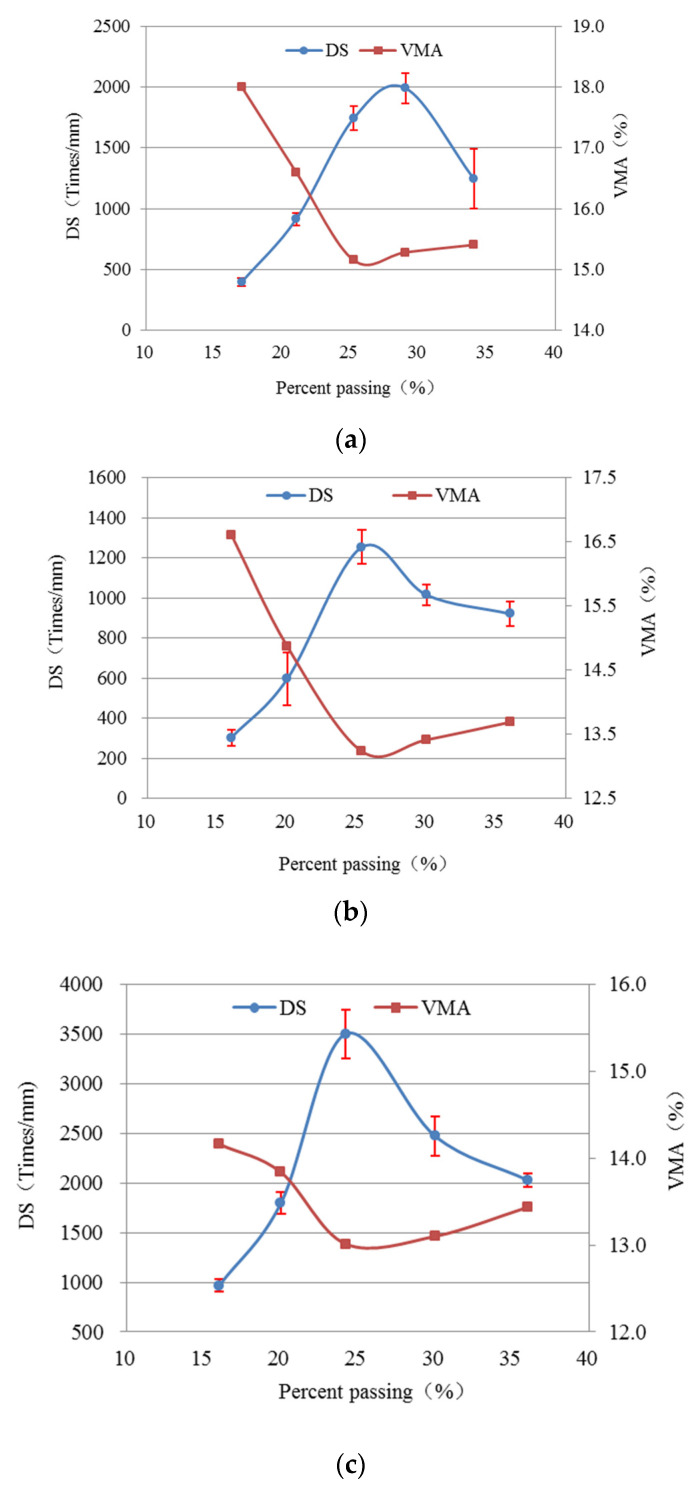
The values of DS and VMA of various AC mixtures versus PP. (**a**) AC-13; (**b**) AC-20; (**c**) AC-25.

Wheel-tracking tests were performed in triplicate for each AC mixture, and the values of DS for AC-25, AC-20 and AC-13 are displayed in Figure 9 (the error bars indicate standard deviation). To perform a comparative analysis of the key parameter (VMA) and RP of the three AC mixtures, the values of VMA are also plotted in Figure 9.

As shown in Figure 9, the values of DS first elevate and then decline with the increasing percentage passing of the BS, which is opposite to the trend of VMA. The DS of different AGs of AC-25, AC-20 and AC-13 have the same rule of change. However, the values of DS are not influenced by the nominal maximum aggregate sizes. In addition, statistical tests were conducted by employing the analysis of variance (ANOVA) method. One-way ANOVA is a commonly used technique for determining the effect of gradation types on the RP of AC mixtures according to the percentage passing of the BS. Table 6 demonstrates the ANOVA results of the DS of AC mixtures. Based on the *F*-statistics and *F*-critical, AG is an important factor affecting the values of DS of the three AC mixtures with a 95% significance level (α = 0.05). It also can be seen that the peak values of DS nearly correspond to the smallest values of VMA (Figure 9). It is speculated that AC mixtures have the best RP when the coarse skeleton structure is in its densest state, which is desirable for the design of the AC mixture. That condition indicates that the rutting resistance of the AC mixture mainly depends on the grading structure of the aggregate. Therefore, it is important to optimize AG for enhancing the high-temperature RP of AC mixtures.

### 3.3. Discussion

As we all know, one of the most defining characteristics of an SMA is the concept of a stone-on-stone skeleton. This is where a large proportion of coarse aggregate particles are in contact with each other to form a skeleton or framework with relatively large voids. The sand-sized particles, filler and binder are then accommodated within the voids in the coarse aggregate skeleton [27]. Supposing that the skeleton of the AC mixture is composed of aggregates with a particle size larger than 2.36 mm, the skeletal dense structure (i.e., stone-on-stone skeleton with the densest state) can be formed as in the case of SMA. Referring to the method for assessing the stone-on-stone aggregate skeleton of SMA, the parameters of VCA and VCA_mix_ can also be applied to determine the skeletal dense structure of the AC mixture. Based on the above experimental results (see Table 5 and Figure 6, Figure 7 and Figure 8), it can be seen that the values of VCA_mix_ (34.37, 35.52 and 36.1 for AC-25, AC-20 and AC-13, respectively) are very close to those of VCA (34.12, 35.65 and 36.01 for AC-25, AC-20 and AC-13, respectively) when the percentage passing of the 2.36 mm sieve is ≈25 for each AC mixture. Moreover, at this percentage passing, the VMA and AV almost have the minimum values, which demonstrate that the coarse skeleton structure is in its dense state. Hence, the skeletal dense structure of the AC mixture can be evaluated by comparing the values of VCA_mix_ and VCA. Additionally, the reference values of the percentage passing of BS are presented for guiding the gradation design of the AC mixture.

## 4. Summary and Conclusions

According to the results and analyses on the VPs and RP of AC mixtures with various AGs, the main findings and conclusions are summarized as follows:

(1) AG has crucial effects on the VPs of AC mixtures. The values of VMA first decline and then elevate with the increasing percentage passing of the BS; those of AV reduce monotonically and appear to be constant, while those of VCA_mix_ show a linear increase.

(2) Statistical analysis of the wheel-tracking test data indicates that AG is important for enhancing the RP of AC mixtures. The values of DS first elevate and then decline with the increasing percentage passing of the BS, which is opposite to the trend of VMA.

(3) AC mixtures show the best RP when the aggregate skeleton structure is in its densest state, which corresponds to the smallest values of VMA. Therefore, it is necessary to optimize AG to enhance the high-temperature RP of AC mixtures.

(4) Based on the experimental results and analyses, the AC mixture can be designed to be a skeletal dense structure, provided that 2.36 mm is employed as the BS between fine and coarse aggregates and the percentage passing of the BS is appropriate. In addition, the skeletal dense structure of the AC mixture can be evaluated by comparing the values of VCA_mix_ and VCA.

(5) This research is focused on the RP of AC mixtures; the effects of AG on moisture susceptibility and fatigue performance will be researched next. Further studies with other asphalt binders are also needed to verify our findings.

## Figures and Tables

**Table 1:** Properties of the basalt coarse aggregates.

Properties	Test Values			Specification
	10–15 mm	5–10 mm	3–5 mm	
Apparent specific gravity	2.835	2.848	2.823	≥2.60
Bulk specific gravity	2.770	2.679	2.703	--
Water absorption (%)	1.0	1.5	1.2	≤2.0
Percent of flat and elongated particles (%)	8.3	9.5	9.8	≤15
Crushed stone value (%)	12.6	12.6	--	≤26
L.A. abrasion (%)	9.3	9.6	9.0	≤28

**Table 2:** Properties of the lime coarse aggregates.

Properties	Test Values				Specification
	20–30 mm	10–20 mm	5–10 mm	3–5 mm	
Apparent specific gravity	2.737	2.727	2.747	2.673	≥2.50
Bulk specific gravity	2.700	2.687	2.694	2.612	--
Water absorption (%)	0.5	0.5	0.7	0.9	≤3.0
Percent of flat and elongated particles (%)	10.3	11.5	9.8	11.0	≤15
Crushed stone value (%)	18.5	18.5	18.5	--	≤28
L.A. abrasion (%)	17.3	17.6	17.2	--	≤30

**Table 3:** Properties of the two fine aggregates.

Properties	Test Values		Specification
	Basalt	Limestone	
Apparent specific gravity	2.821	2.723	≥2.50
Angularity (s)	38	36	≥30

**Table 4:** Properties of the Pen 70 asphalt binder.

Index	Softening Point (°C)	Penetration (25 °C, 0.1 mm)	Specific Gravity (15 °C)	Ductility (cm)	
				15 °C	10 °C
Test values	49.6	71	1.021	>100	40

**Table 5:** Results of the vibrating compaction test of coarse aggregates.

AC Mixes	Proportions of Different Coarse Aggregates					VCA (%)
	20–30 mm	10–20 mm	10–15 mm	5–10 mm	3–5 mm	
AC-13	--	--	29	51	20	36.01
AC-20	--	65	--	20	15	35.65
AC-25	25	45	--	15	15	34.12

**Table 6:** ANOVA results for the DS of AC mixtures.

Source of Variation	Degree of Freedom	Sum of Squares	Mean Square	*F*- Statistics	*F*- Critical	*p*- Value
AC-13						
Gradation	4	4,885,150	1,221,288	69.83495	3.47805	2.87 × 10^−7^
Error	10	174,882	17,488.2			
Total	14	5,060,032				
AC-20						
Gradation	4	1,656,796	414,198.9	64.31926	3.47805	4.26 × 10^−7^
Error	10	64,397.33	6439.733			
Total	14	1,721,193				
AC-25						
Gradation	4	10,371,596	2,592,899	107.8359	3.47805	3.52 × 10^−8^
Error	10	240,448.7	24,044.87			
Total	14	10,612,044				

## Data Availability

The data presented in this study are available on request from the corresponding author. The data are not publicly available due to them forming part of an ongoing study.
